# Pseudouridine synthase 1 promotes progression of hepatocellular carcinoma via *mTOR* and *MYC* signaling pathways

**DOI:** 10.3389/fonc.2025.1576651

**Published:** 2025-03-18

**Authors:** Li Chen, Yonghuang Tan, Weinan Li, Lunkai Huang, Kang Li, Zanjie Feng, Cijun Peng, Yong Mei

**Affiliations:** ^1^ Diagnostics Laboratory, Affiliated Hospital of Zunyi Medical University, Zunyi, Guizhou, China; ^2^ Department of Gastrointestinal Surgery, The First Affiliated Hospital of Sun Yat-sen University, Guangzhou, China; ^3^ Department of Hepatobiliary Surgery, The Affiliated Hospital of Guizhou Medical University, Guiyang, Guizhou, China

**Keywords:** pseudouridine synthases 1, hepatocellular carcinoma, MYC, mTOR, therapeutic target

## Abstract

Pseudouridine synthases (PUSs) are associated with the development and progression of various cancers. However, the role of pseudouridine synthase 1 (PUS1) on HCC is unclear. The purpose of this study is to explore the biological role and mechanism of PUS1 in HCC growth and progression. We identified the expression of PUS1 in HCC. The biological roles and downstream cell signaling pathways of PUS1 were explored to clarify the molecular mechanism of PUS1 in the growth and development of HCC. The results showed that the expression of PUS1 was correlated with HCC progression, metastasis, and poor survival. In addition, the knockdown of PUS1 dramatically inhibited cell proliferation and colony formation and promoted cell apoptosis. GSEA analysis revealed that c-MYC, DNA repair, and mTORC1 pathways were significantly enriched in patients with high PUS1 expression. An intersection of the PUS1-dependent Ψ modification genes and c-MYC or mTORC1 pathway genes was performed. The expression of a part of these genes changed after PUS1 knockdown. Meanwhile, the expression of c-MYC and mTOR were down-regulated after PUS1 knockdown, but the inhibitory effect of PUS1 on cell growth capacity was not enhanced after inhibiting c-MYC or mTOR pathways. In conclusion, PUS1 regulates the occurrence and development of HCC through c-MYC and mTOR-related signaling pathways. It could be a novel molecule for clinical diagnosis, progression surveillance, prognosis assessment and therapeutic target of HCC.

## 1 Introduction

Hepatocellular carcinoma (HCC), the most common type of liver cancer, is the third leading cause of cancer death worldwide, with a relative 5-year survival rate of approximately 18% (1). Several factors may increase the risk of HCC, including chronic hepatitis B and hepatitis C, alcohol dependence, metabolic liver disease (especially nonalcoholic fatty liver disease), and dietary toxins (2–4). Unresectable HCC patients still face unmet medical needs and a poor prognosis (5, 6). Early diagnosis and timely treatment of HCC are the most fundamental solution to improve the prognosis of patients. Currently, abdominal ultrasound is the standard screening test used in clinical practice, but its sensitivity is only around 50% (7). In patients with obesity, ultrasonography becomes even less sensitive. A few HCC biomarkers with a real clinical effect have emerged. Alpha-fetoprotein (AFP) has been used for more than 60 years. However, a recent study showed that AFP is insufficiently sensitive to HCC (8).Elevated serum des-gamma-carboxy prothrombin (DCP) expression is associated with HCC and poor prognosis (9). However, current limitations for the early diagnosis of HCC highlight the need for more effective HCC surveillance tests.

Pseudouridylation (Ψ) is the most abundant and widespread type of RNA epigenetic modification in living organisms, and the Ψ of the noncoding RNAs of the translation and splicing machineries is important for their functions (10). However, the biological role of Ψ remains poorly understood.

In yeast, most mRNA pseudouridines have been genetically assigned to two conserved pseudouridine synthases (PUSs), i.e., PUS1 and PUS7 (11), which are nuclear-localized during normal growth. Human PUSs localize to the nucleus or have nuclear isoforms (12) and are active in the nucleus, where they target pre-mRNA (13). Previous studies on PUSs associated human diseases are primarily focus on the PUS7 and dyskerin pseudouridine synthase 1 (DKC1). PUS7 plays a critical role in development and brain function as a versatile RNA modification enzyme targeting many RNAs (14). DKC1 is markedly upregulated in many different human cancer tissues, including HCC and colorectal cancer, which impacts the overall survival and progression-free survival outcomes of patients (15, 16). Recent results for a rapid, high-throughput *in vitro* assay to quantitatively assess Ψ of thousands of sequences in parallel validated 83% of mRNA Ψ genetically assigned to yeast PUS1 *in vivo*. Unfortunately, these studies failed to detect Ψ of some known human PUS1 tRNA targets *in vitro* (17). Despite the latest research reported that PUS1 promotes HCC through mRNA pseudouridylation to enhance the translation of oncogenic mRNAs (18), the biological role and mechanism of PUS1 remain poorly understood, especially in human cancer.

Herein, the objectives of this study are to (1) investigate the prognostic value of PUS1 gene expressions in HCC using datasets from The Cancer Genome Atlas (TCGA), the Clinical Proteomic Tumor Analysis Consortium (CPTAC), the UALCAN website, THE HUMAN PROTEIN ATLAS, the TCGA-HCC database, and HCC patients’ tumor tissues; (2) investigate the potential mechanism of PUS1 affecting occurrence and development of HCC; and (3) verify the mechanism based on experiments on HCC cell lines. This study provides a potential novel biomarker and therapeutic target for improving clinical diagnosis, progression surveillance, and prognosis assessment of HCC.

## 2 Materials and methods

### 2.1 The expression analysis of PUSs in HCC

TGCA_HCC database (https://xena.ucsc.edu/) was used to confirm the mRNA expression of 13 PUSs. CPTAC database (https://cptac-data-portal.georgetown.edu/) was used to confirm the protein expression of 12 PUSs (Project: Integrated Proteogenomic Characterization of HBV-related HCC).

### 2.2 PUS1 expression analysis in HCC

UALCAN (http://ualcan.path.uab.edu/) was used to analyze the mRNA (TCGA module) and protein (CPTAC module) expression for PUS1. The PUS1 expression based on sample types, individual cancer stage, and tumor grade was analyzed in “Expression” module. Additionally, the PUS1 expression in E-MTAB-6695, E-MTAB-4171, GSE39791, GSE47197, GSE54236, GSE25079, E-MTAB-8887, GSE17548, GSE56140, and GSE54238 databases based on sample types, liver disease, and tumor grade was extracted from ArrayExpress (https://www.ebi.ac.uk/arrayexpress/, accessed on 25 August 2022).

### 2.3 The IHC analysis of PUS1 in THE HUMAN PROTEIN ATLAS

The immunohistochemistry (IHC) staining and subcellular localization analysis of PUS1 in HCC were obtained from THE HUMAN PROTEIN ATLAS (https://www.proteinatlas.org/) TISSUE and PHATHOLOGY module. ImageJ was used for quantitative analysis.

### 2.4 Survival analysis in Kaplan–Meier plotter

Kaplan–Meier plotter (http://kmplot.com/analysis/) was used to assess the correlation between the expression of 30,000 genes and patient survival. The overall survival (OS), disease-specific survival (DSS), progression-free survival (PFS), and relapse-free survival (RFS) curves of 13 PUSs were analyzed. The high and low PUSs expression groups were defined as above or below the median expression value of the 13 PUSs in Kaplan–Meier Plotter website “using multiple genes” module (17). The expression cutoff was split by “auto select best cutoff” option. When this checkbox was selected, all possible cutoff values between the lower and upper quartiles were computed, and the best performing threshold was used as a cutoff. After excluding the biased arrays, the Kaplan–Meier survival curves were obtained. Using the “restrict analysis to subtypes” option, correlation of PUS1 mRNA expression and clinical prognosis in HCC with different clinicopathological factors was obtained.

### 2.5 GSEA analysis

TGCA_HCC databases (https://xena.ucsc.edu/) were downloaded and mined. Data from patients with the top 30 tumors with the highest PUS1 expression and the bottom 30 tumors with the lowest PUS1 expression in TCGA_HCC were used for gene set enrichment analysis (GSEA) using hallmark gene sets.

### 2.6 Cell culture and transfection

LO2, SNU449, HepG2, and PLC/PRF/5 cell lines were purchased from the American Type Culture Collection (ATCC; Manassas, VA, USA). Cells were cultured and maintained in Dulbecco’s modified Eagle medium (DMEM); supplemented with 10% fetal bovine serum(FBS), 100 U/mL penicillin, and 100 µg/mL streptomycin. Cells were incubated in a humidified chamber at 37°C under 5% CO_2_.SNU449 and HepG2 cell lines were transfected by PUS1–siRNA (siPUS1#1: GCCAGAGCTTCATGATGCA; siPUS1#2: GTCGGGTCCTCACAATTCA; negative control (NC): TTCTCCGAACGTGTCACGT, from RIBOBIO (China)). The transfection was performed according to the protocol (Beyotime, lp8000, C0533-1.5 ml, Shanghai, China).

### 2.7 Antibodies

Several antibodies were obtained, including anti-PUS1 (EPR13235(B); Abcam), anti-mTOR (66888-1-1g; Proteintech; Rosemont, IL, USA), anti-c-Myc (9402; Cell Signaling Technology; Danvers; MA, USA), Phospho-S6 Ribosomal Protein (Ser235/236) (2211S; Cell Signaling Technology; Danvers; MA, USA) and anti-GAPDH antibodies (AB9132; Promega Corporation; Madison, WI, USA). Goat anti-rabbit Alexa Fluor 488 (A-31566) and goat anti-mouse Alexa Fluor 647 (A-21242) were used as the secondary antibodies.

### 2.8 Cell viability assay

Cell viability was evaluated using classical MTT assay. Cell cultures with a density of 3,000 cells/well were cultured in 96-well plates for 24 h. Cells were transfected with siPUS1 and NC. After being cultured for 72 h and washed thrice with phosphate buffered saline (PBS) buffer, the cells were treated with MTT (5 mg/mL) for 4 h. The formazan was dissolved in 150 μL of dimethyl sulfoxide (DMSO) after removal of supernatants. A microplate reader (Biotek Cytation5) was used for colorimetric measurements at 490 nm wavelength.

### 2.9 Colony forming assay

Adherent cells were transfected with siPUS1 and NC in 6-well plates (500−1,000 cells/well). Following incubating without changing the cell culture medium for 14 days, crystal violet staining (0.1%) was performed after 15 min of fixation with 4% paraformaldehyde. Results were visualized by camera.

### 2.10 Western blotting

Adherent cells were transfected with siPUS1 and NC in 24-well plates (2 × 10^4^ cells/well). Following incubating without changing the cell culture medium for 72 h, protein lysates were collected with radio-immunoprecipitation assay (RIPA) buffer (Sangon; Shanghai, China). The protein concentration was determined by the ThermoFisher Scientific BCA protein assay kit (NCI3225CH). A total of 20 μg of total proteins from supernatant was analyzed on sodium dodecyl-sulfate polyacrylamide gel electrophoresis (SDS-PAGE), and then transferred to nitrocellulose membrane. Following blocking with 5% non-fat milk diluted in 1× Tris-buffered saline with 0.1% Tween-20 (TBST) buffer for 1 h, these nitrocellulose membranes were incubated with primary antibodies at 4°C overnight. Subsequently, the nitrocellulose membrane was washed by TBST, and then incubated with an HRP-conjugated secondary antibody (1:5000) at room temperature for 1.5 h. The protein bands were visualized by enhanced chemiluminescence (ECL) reagents and chemiluminescence system (GE Amersham Imager 600; Boston, MA, USA).

### 2.11 Flow cytometric analysis of Annexin V apoptosis assay

Apoptotic and necrotic cells, following treatment, were detected by the Annexin V-FITC Apoptosis Detection Kit (No. K101-25; BioVision). A total of 5 × 10^5^ cells were cultured in 6-well plates, and then they were transfected with siPUS1 and NC, followed by incubation for 72 h without changing the cell culture medium. A total of 1 × 10^5^ cells were collected and resuspended in 500 μL, and then the binding buffer of 5 μL of Annexin V-FITC and 5 μL of propidium iodide (PI) was added, which was incubated at room temperature in the dark for 5 min. Annexin V-FITC binding was analyzed by flow cytometry (excitation, 488 nm; emission, 530 nm).

### 2.12 IHC staining

The immunohistochemical (IHC) method was used to detect the expression of PUS1 protein in 45 pairs of paraffin-embedded HCC tissues and matched adjacent normal liver tissues. The slices were heated, dewaxed, rehydrated, and put into sodium citrate buffer (pH buffer = 6. 0) for antigen repair. The slide was then soaked in 3% hydrogen peroxide to inhibit endogenous peroxidase activity and sealed with sheep10% FBS/PBS. After rinsing three times, The slides were incubated with primary antibody against PUS1 (1:100; AB175240; Abcam; Cambridge, UK) overnight at 4°C. Slices were washed three times using PBS and then treated with a second antibody (anti-rabbit Ig GJI 1D 2000 diluted, # 7074; Cell signal, Danvers, MA, USA) for 40 min at 37°C. After being stained with 3BI-3-diaminobenzidine (DAB), it was stained with hematoxylin, dehydrated, sealed, and observed.

The IHC staining scores were evaluated by two pathologists blinded to clinical materials. A quick scoring system from 0 to 12 that combined the intensity and percentage of the positive signal was used. About the intensity of, 0, 1, 2, and 3 represented no staining, weak staining, intermediate staining, and strong staining, respectively. According to the percentage of the positive staining, the staining degree is scored as 0 (0), 1 (1%–25%), 2 (26%–50%), 3 (51%–75%), and 4 (76%–100%). The score of the intensity and range of an image was used as the final score of PUS1 (0–12). Tissue protein expression was defined as high level when the score was ≥7 and low level when the score was ≤6.

### 2.13 RT-PCR

RNA extraction with Trizol (Invitrogen; Carlsbad, CA, USA) and real-time (RT)-PCR was performed for gene expression. All the reactions were performed with Takara SYBR Premix Ex Taq (Takara; Dalian, Liaoning, China) and quantified by a CFX96 Real-Time PCR System (Bio-Rad) for the qPCR-based mRNA export analysis. The 2(-Delta Delta C (T)) method was used for calculating the relative fold changes in cytoplasmic/nuclear ratios. The primer pairs used for qPCR are listed in **Supplementary Material** (**Supplementary Table S1**).

### 2.14 Statistical analysis

Student’s T-test and one-way analysis of variance (ANOVA) test were performed to analyze the difference between two groups and among more than two groups, respectively. Pearson analysis was performed for correlation analysis. Data were presented as the mean ± standard error of mean, and *P* < 0.05 was considered statistically significant.

## 3 Results

### 3.1 PUSs expression increased in HCC and predicted poor prognosis

**Figure 1:**
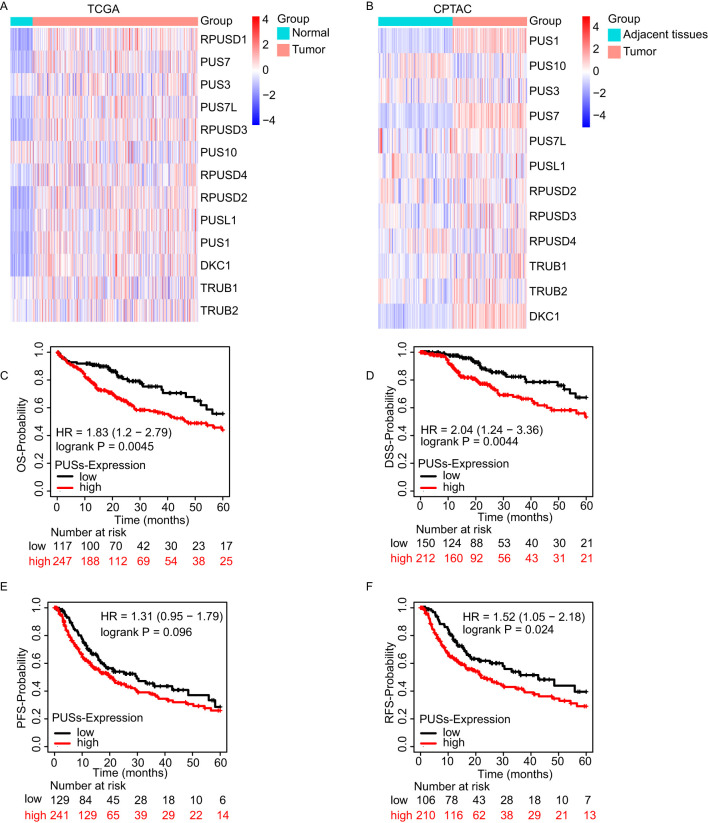
PUSs expression increased in HCC and predicted poor prognosis. **(A)** The expression of 13 PUSs in HCC between cancer tissue and adjacent tissue based on TCGA database. **(B)** The expression of 12 PUSs in HCC between cancer tissue and adjacent tissue based on CPTAC database. **(C–F)** The OS, DSS, PFS, and RFS Kaplan–Meier survival curves comparing the high and low expression of 13 PUSs in HCC patients.

The mRNA expression of most PUSs, such as *RPUSD1*, *PUS7, PUS7L, RPUSD3, RPUSD2, PUSL1, PUS1*, and *DKC1* (**Figure 1A**), and protein expression of PUS1, PUS7, and DKC1 were significantly increased in tumors (**Figure 1B**). The results from Kaplan–Meier Plotter website demonstrated that HCC patients with high PUSs expression predicted shorter OS, DSS, and RFS time (**Figures 1C–F**). The detail of survival analysis on the 13 PUSs is shown in **Supplementary Material** (**Supplementary Table S2**). In summary, most PUSs expression increased in HCC, and high PUSs expression predicted shorter OS, DSS, and RFS time.

### 3.2 PUS1 expression increased in HCC

**Figure 2:**
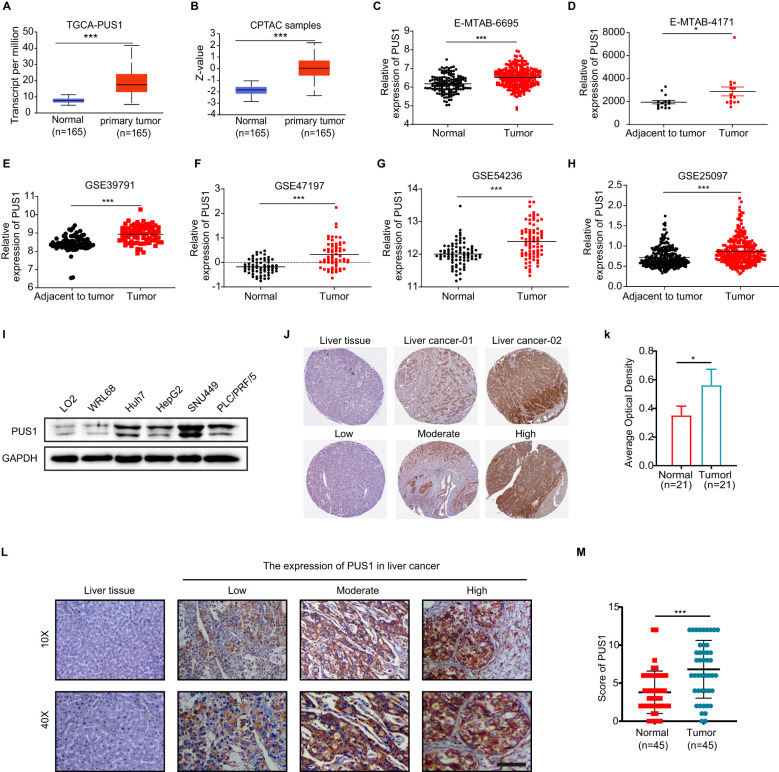
PUS1 expression increased in HCC. **(A)** The mRNA expression of PUS1 between normal tissue and primary HCC tissue analyzed based on the UALCAN website. **(B)** The protein expression of PUS1 between normal tissue and primary HCC tissue analyzed based on the CPTAC database in UALCAN website. **(C–H)** The mRNA expression of PUS1 between normal tissue and primary HCC tissue analyzed based on E-MTAB-6695, E-MTAB-4171, GSE39791, GSE47197, GSE54236, and GSE25079. **(I)** The protein expression of PUS1 between normal liver cell lines (LO2, WRL68) and HCC cell lines (Huh7, HepG2, SUN449, and PLC/PRF/5). **(J, K)** The immunohistochemistry of PUS1 in liver tissue and HCC from THE HUMAN PROTEIN ATLAS. **(L, M)**. The expression of PUS1 between liver tissue and HCC from 45 patients. (*P < 0.05, ***P < 0.001).

It was found that PUS1 expression was increased in HCC in the TCGA and CPTAC datasets (**Figures 2A, B**). To further confirm the high expression of PUS1 in HCC tissues, RNA sequencing data of HCC and normal liver tissues were obtained from ArrayExpress website. Six datasets were analyzed and all the results showed that PUS1 expression was increased in tumor tissues (**Figures 2C–H**). It was also observed that PUS1 expression was increased in HCC cell lines, including Huh7, HepG2, SUN449, and PLC/PRF/5, compared with normal cell lines LO2 and WRL68 (**Figure 2I**). Moreover, results based on the immunohistochemically staining of PUS1 in cancer and normal tissues of HCC patients in THE HUMAN PROTEIN ATLAS database showed that PUS1 was highly expressed in HCC patients (**Figures 2J, K**). Meanwhile, immunohistochemical staining was performed on 45 pairs of tissues (tumors and normal liver tissues) collected from the HCC patients of our hospital, and the results consistently suggested that PUS1 was elevated in liver tumor tissues (**Figures 2L, M**). In short, PUS1 is highly expressed in HCC and may be a novel biomarker for HCC.

### 3.3 High PUS1 expression was positively correlated with occurrence and progression of HCC

**Figure 3:**
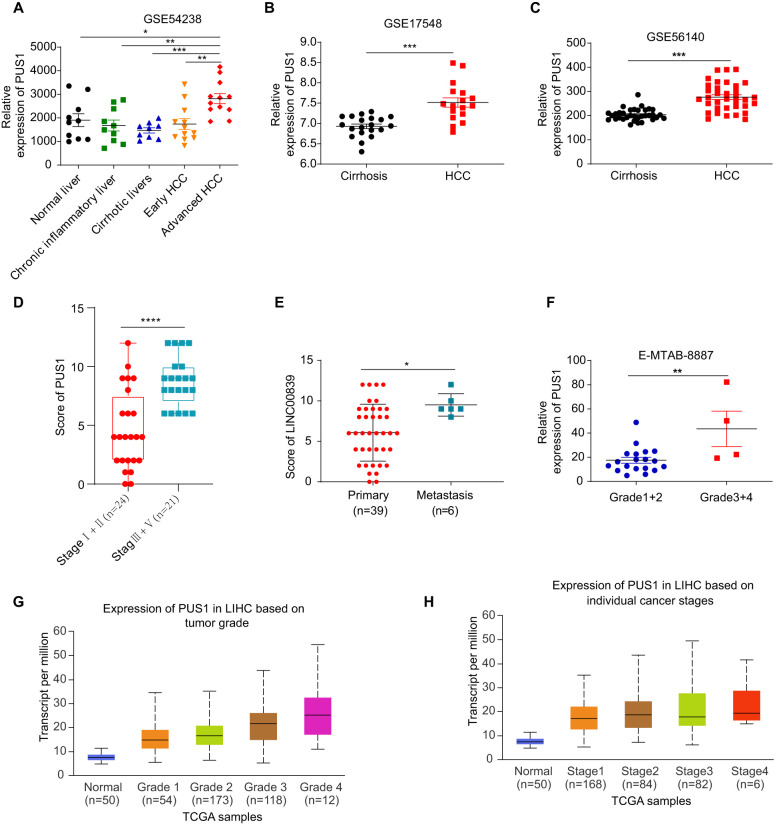
High PUS1 expression was positively correlated with occurrence and malignant progression of HCC. **(A)** The expression of PUS1 stratified by normal, hepatitis, cirrhosis, and HCC based on the GSE54238. **(B, C)** The expression of PUS1 stratified by cirrhosis and HCC based on the GSE17548 and GSE56140. **(D)** The expression of PUS1 based on different stage in 45 HCC patients. **(E)** The expression of PUS1 in primary and metastasis tumor in 45 HCC patients. **(F)** The expression of PUS1 stratified by different grade in HCC based on the E-MTAB-8887. **(G)** The expression of PUS1 stratified by different stage in HCC based on the UALCAN website. **(H)** The expression of PUS1 stratified by different grade in HCC based on the UALCAN website. (*P<0.05, **P<0.01, ***P<0.001, ****P<0.0001).

Subgroup analysis of PUS1 expression in HCC based on the ArrayExpress database was performed. The expression of PUS1 was increased in tumor compared to hepatitis or cirrhosis (**Figures 3A–C**). The PUS1 expression in HCC tissues collected from patients with different stages and metastasis status proved that PUS1 expression was higher in patients with stage III/IV than in patients with stage I/II(**Figures 3D, F–H**), and PUS1 expression was higher in patients with metastasis than in primary tumors (**Figure 3E**). The expression of PUS1 in HCC was positively correlated with tumor stemness (**Supplementary Figure S1**). These results indicated that high expression of PUS1 was related to the occurrence and malignant progression of HCC.

### 3.4 High PUS1 expression in HCC predicted poor prognosis

**Figure 4:**
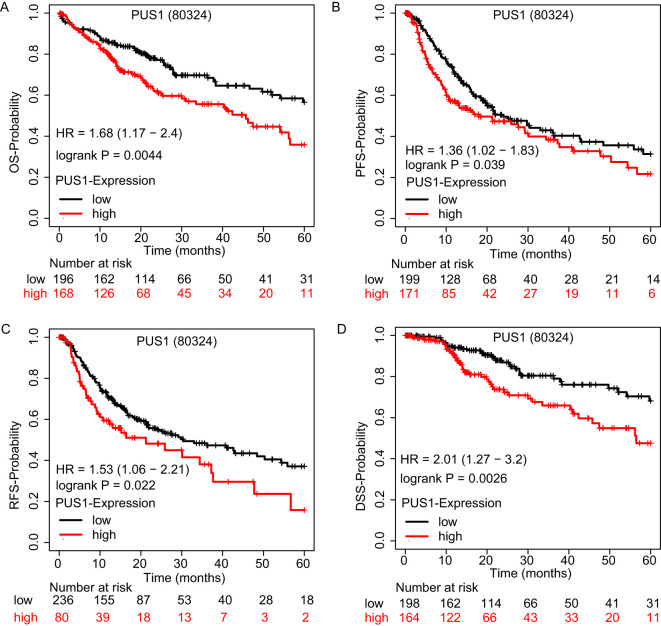
High PUS1 expression in HCC predicted poor prognosis. **(A–D)** OS, DSS, PFS, and RFS Kaplan–Meier curves comparing the high and low expression of PUS1. (80324 is the RNA-seq ID of PUS1.).

HCC patients were divided into high- and low-expression groups based on the best cutoff value in Kaplan–Meier Plotter website. The relationship between the PUS1 expression and clinical characteristics of HCC patients is shown in **Supplementary Excel** (**Excel 1**). HCC patients with high PUS1 expression had a shorter OS time (**Figure 4A**). Furthermore, high PUS1 expression predicted shorter PFS, RFS, and disease-specific survival time (**Figures 4B–D**), and the detail of PUS1 survival analysis is shown in the **Supplementary Material** (**Supplementary Table S3**), indicating that PUS1 was related to the malignant progression. Overall, high expression of PUS1 predicted a poor prognosis in HCC patients.

### 3.5 PUS1 silencing inhibited HCC cell proliferation and colony formation, and promoted cell apoptosis

**Figure 5:**
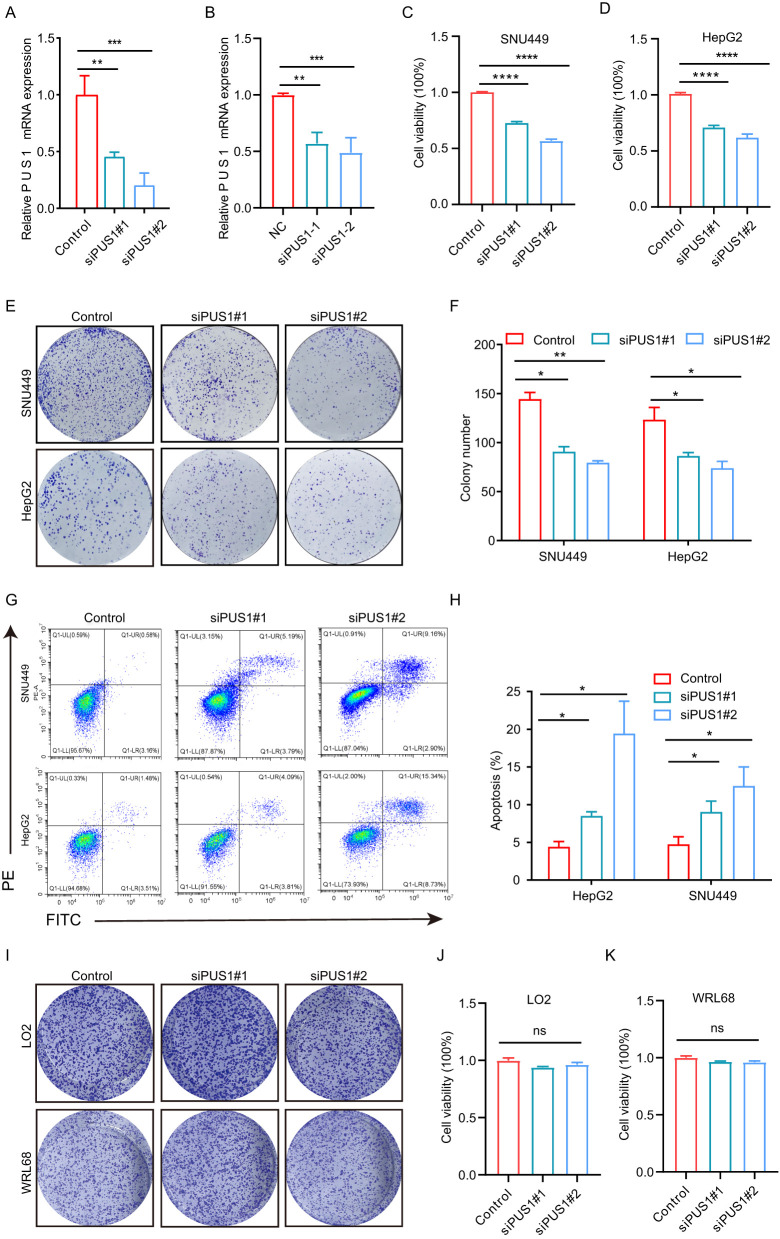
PUS1 silencing inhibited HCC cell proliferation and colony formation, and promoted cell apoptosis. **(A)** RT-PCR analysis of PUS1 expression in SNU449 cell and HepG2 **(B)** after transfection with control or PUS1 siRNAs. **(C, D)** Cell proliferation of SNU449 and HepG2 cells transfected with NC or PUS1 siRNA analyzed by MTT assay. **(E, F)** Knockdown of PUS1 dramatically inhibited the cell colony formation ability. **(G, H)** Apoptosis assay by flow cytometric analysis revealed PUS1–siRNA promoted apoptosis in SNU449 and HepG2 cells. **(I)** Knockdown of PUS1 hardly inhibited the cell colony formation ability of LO2 and WRL68 cells. **(J, K)** Knockdown of PUS1 hardly inhibited the cell proliferation of LO2 and WRL68 cells. (*P<0.05, **P<0.01, ***P<0.001, ****P<0.0001; ns, P>0.05).

To explore the functional role of PUS1 in the malignant behavior of HCC cells, the expression of PUS1 in HCC cells was knocked down using two different siRNAs targeting PUS1 (siRNA#1 and siRNA#2) (**Figures 5A, B**). MTT assay showed that PUS1 siRNA could suppress the growth in SNU449 and HepG2 cells (**Figures 5C, D**). Colony formation assay demonstrated that SNU449 and HepG2 cells treated with PUS1 siRNA generated fewer colonies than the NC group (**Figures 5E, F**). Apoptosis assay by flow cytometric analysis revealed that PUS1 siRNA promoted apoptosis of SNU449 and HepG2 cells (**Figures 5G, H**). In contrast, PUS1 knockdown did not inhibit cell growth and colony formation in normal liver cell lines (LO2, WRL68) (**Figures 5I–K**). In summary, PUS1 is an important target that promotes the proliferation of HCC cells.

### 3.6 PUS1 promoted tumorigenesis and progression of HCC dependent on the mTOR and MYC pathways

**Figure 6:**
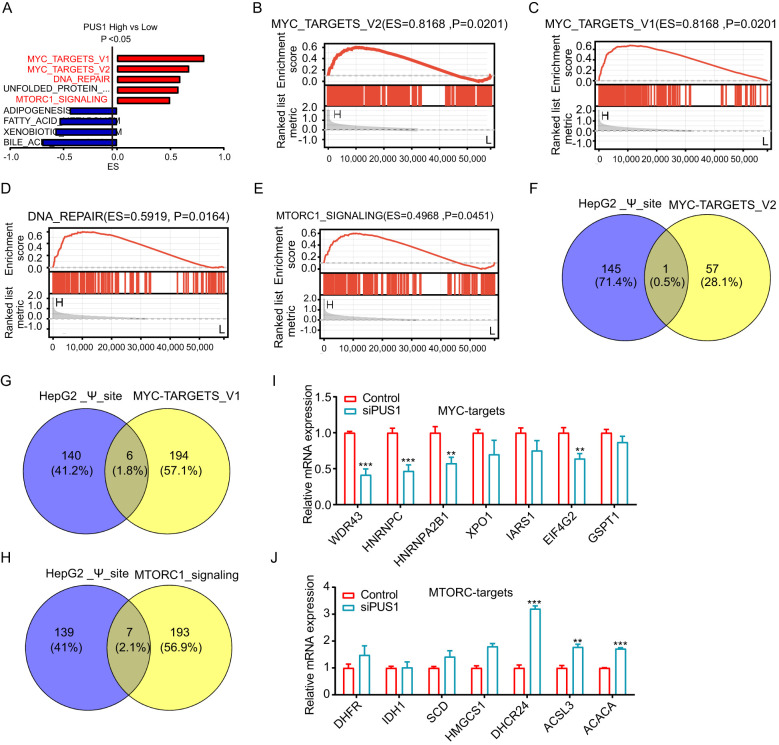
PUS1 promoted tumorigenesis and progression of HCC closely related to the mTOR and MYC pathways. **(A)** GSEA analysis between high and low PUS1 expression patients using hallmark gene sets based on TCGA–HCC database. **(B–E)** GSEA analysis revealed that MYC pathways, DNA repair, and MTORC1 pathways were significantly enriched in patients with high PUS1 expression. **(F–H)** The intersection analysis of the PUS1–dependent Ψ modification genes and MYC or MTORC1 pathway genes were applied. **(I, J)** RT–PCR analysis of PUS1 potential downstream targets in HepG2 cell after transfection with control or PUS1 siRNA. (**P<0.01, ***P<0.001).

To better understand the potential mechanisms and the downstream targets of PUS1 in HCC, GSEA analysis between high and low PUS1 expression patients using hallmark gene sets based on TCGA_HCC database was performed. Results indicated that MYC pathways, DNA repair, and MTORC1 pathways are the potential downstream pathways (**Figures 6A–E**). In order to determine the downstream potential targets of PUS1 regulating MYC or MTORC1 pathways, an intersection of the PUS1-dependent Ψ modification genes and MYC or MTORC1 pathway genes was performed. A total of 14 genes were found **Supplementary Excel** (**Excel 2**). These genes might be potential downstream targets of PUS1 (**Figures 6F–H**). After knocking down PUS1, the expression of potential downstream oncogenic genes of MYC pathway, including WDR43, EIF4G2, HNRNPC, and HNRNPA2B1, were down-regulated (**Figure 6I**). Moreover, the expression of potential downstream tumor suppressor genes of MTORC1 pathway, including ACSL3, DHCR24, and ACACA, were increased after knocking down PUS1 (**Figure 6J**).

**Figure 7:**
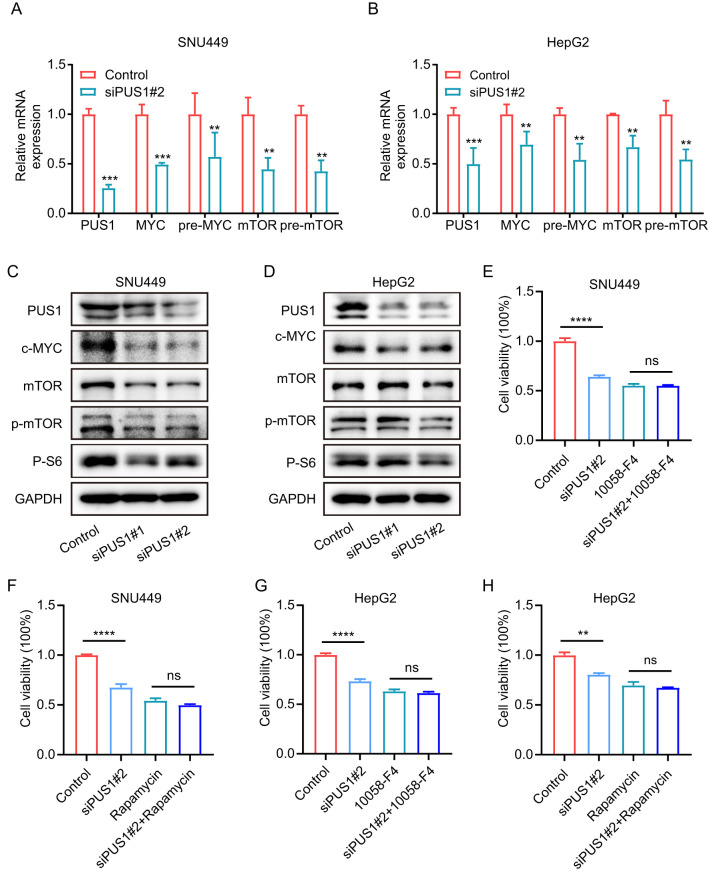
PUS1 promotes cell proliferation depending on the mTOR and MYC pathways. **(A, B)** RT–PCR analysis of the mRNA expression of MYC, pre-MYC, mTOR and pre-mTOR in HCC cells after transfection with control or PUS1 siRNA. **(C, D)** WB testing of PUS1, MYC, mTOR, p-mTOR and P-S6 expression in HCC cells after transfection with control or PUS1 siRNA. **(E-H)** Cell proliferation of SNU449 and HepG2 cells transfected with PUS1 siRNA treatment with or without inhibitors of MYC (10058-F4, 10μM) or mTOR (rapamycin, 200nM) were analyzed by MTT assay. (**P<0.01, ***P<0.001, ****P<0.0001; ns, P>0.05).

**Figure 8:**
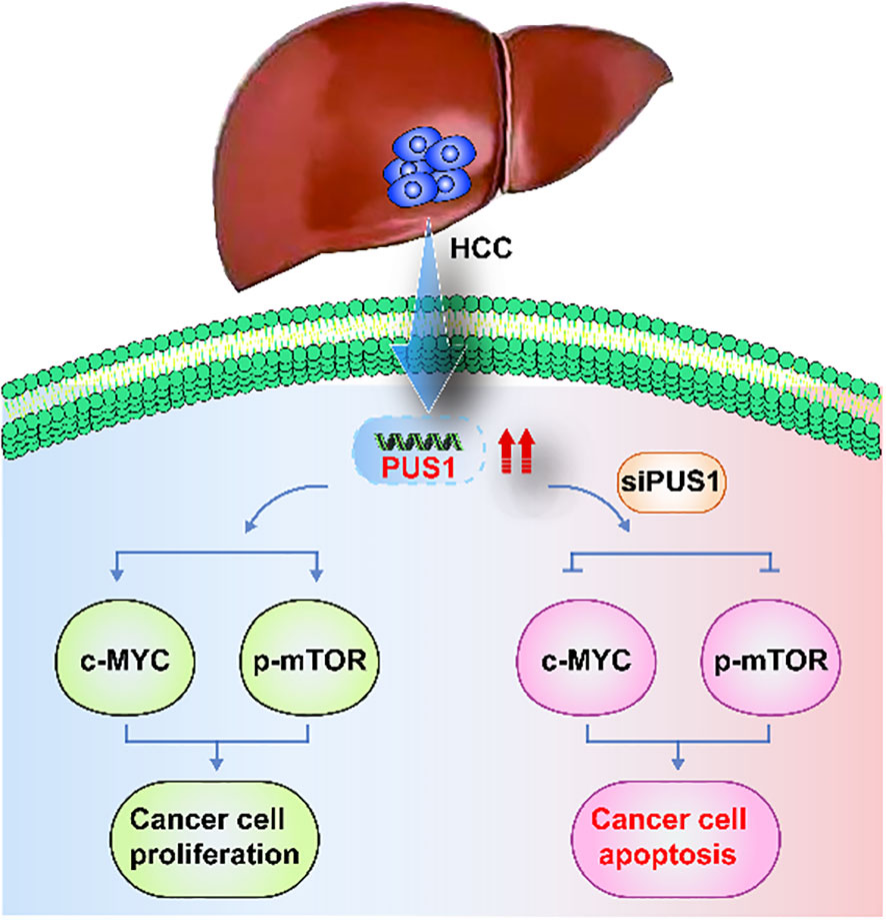
A schematic view of PUS1 promoted tumorigenesis and progression of HCC dependent on the mTOR and MYC pathways.PUS1 expression was increased in HCC. However, knocking down PUS1 in HCC cells, the expression of MYC, p-mTOR and mTOR pathway‐related protein P-S6 were decreased. These results suggested that the role of PUS1 promoted tumorigenesis and progression of HCC is dependent on the mTOR and MYC pathways.

Then, we investigated whether PUS1 promoted tumorigenesis and progression of HCC were dependent on the mTOR and MYC pathways. As expected, knocking down PUS1 in SNU449 and HepG2 cells, the mRNA expression of MYC, pre-MYC, mTOR and pre-mTOR were significantly down-regulated (**Figures 7A, B**). Meanwhile, Western blotting demonstrated that the expression of MYC, p-mTOR and mTOR pathway‐related protein P-S6 were decreased in SNU449 and HepG2 cells (**Figures 7C, D**). In addition, to explore the impact of MYC and mTOR on PUS1‐mediated cell proliferation, SNU449 and HepG2 cells with PUS1 knockdown were treated with inhibitors of MYC (10058-F4) or mTOR (rapamycin) against MYC and mTOR pathways. It was found that the inhibitory effect of PUS1 on cell growth capacity was not enhanced after MYC or mTOR pathways inhibition (**Figures 7E–H**). Altogether, these results suggested that the role of PUS1 promoted tumorigenesis and progression of HCC is dependent on the mTOR and MYC pathways (**Figure 8**).

## 4 Discussion

Our results showed that the expression of most PUSs is increased in HCC tissues, indicating that high PUSs expression predicted a worse prognosis. According to previous studies, Ψ plays a key regulatory role in the development and progression of various human cancers (19). Ψ has been associated with tumorigenesis via dysregulation of Ψ installation machinery (20). For example, high PUS7 expression levels are associated with poor survival in patients with glioblastoma (21); Ψ in difficult-to-treat subsets of MDS is characterized by high risk of progression to acute myeloid leukaemia (22); miR-10b modulates U6 N-6-adenosine Ψ promoting glioblastoma tumorigenesis (23); loss of NOP10 and subsequent reduction in H/ACA box snoRNAs and rRNA Ψ inhibited lung cancer tumorigenesis (24); lacking SNORA24-guided Ψ is associated with liver cancer tumorigenesis (25). Hence, abnormal expression of PUSs can catalyze the formation of Ψ and play an important regulatory role in HCC growth and progression.

Our result indicated that PUS1 expressed significantly higher in cancer tissues than adjacent liver tissues of HCC patients, in line with the results of the patient data analysis of THE HUMAN PROTEIN ATLAS database. It was also found that mRNA expression of PUS1 was increased in HCC tumor tissues compared with normal liver tissues in the UALCAN website, E-MTAB-6695, E-MTAB-4171, GSE39791, GSE47197, GSE54236, and GSE25079. In addition, protein expression of PUS1 in different HCC cell lines was also higher than in normal liver cells. Therefore, PUS1 is highly expressed in liver cancer patients and may be a novel diagnostic biomarker. Through subgroup analysis, it was found that expression of PUS1 was abnormally elevated in tumor compared to hepatitis or cirrhosis; it was higher in patients with stage III/IV than in patients with stage I/II, and it was also higher in patients with metastatic tumors than in primary tumors. These results indicated that PUS1 was related to malignant progression of HCC. The onset and progression of HCC is a multistep process (26), and liver cirrhosis often occurs before live cancer formation (27), no matter whether it is caused by alcoholic hepatitis cirrhosis (28) or by viral hepatitis cirrhosis (29). Based on this result, in clinical practice, PUS1 detection is recommended for patients with chronic liver disease to be a biomarker or a supplementary detection for APF biomarker in early and definite diagnosis for the HCC patients who cannot be clearly identified by imaging examination. However, single biomarker has suboptimal performance for early HCC detection, likely related to tumor heterogeneity. We speculate that the detection of combined AFP with PUS1 possible produce an algorithm with better sensitivity for HCC patients with cirrhosis. Furthermore, the function of PUS1 to the malignant behavior of HCC cells was explored to verify the relevant results. It can be observed in *in vitro* experiments that PUS1 silencing inhibited HCC cell proliferation and colony formation, and promoted cell apoptosis. In summary, our study revealed that PUS1 is a potential novel tumor biomarker and effective therapeutic target for improving clinical diagnosis, progression surveillance, and prognosis assessment of HCC.

In this study, we demonstrated that PUS1 may regulate the occurrence and development of HCC via mTOR and MYC pathways. The potential mechanisms of PUS1 in HCC were explored by GSEA analysis, given that gene expression of MYC pathways, DNA repair, and mTORC1 pathways were significantly changed. PUS1 may promote HCC by regulating MYC and mTOR; after knocking down PUS1 in SNU449 cell, the protein expression of c-MYC and mTOR were significantly down-regulated, with the changing of downstream gene mRNA expression associated with the two pathways. Previous studies have shown that Ψ modification in RNA can affect the mRNA stability, protein translation, and pre-RNA splicing process in cancer (30). The expression of other PUSs, such as DKC1 and PUS7, were closely related to the expression of MYC, but the detailed mechanism is unclear (31). We found that knockdown of PUS1 reduced the expression of MYC and mTOR. The potential mechanism between PUS1 and MYC, and mTOR may regulate through mRNA stability, protein translation, and pre-RNA splicing process. In addition, there are Ψ sites on the CDS region of MYC mRNA in human or mouse, but there are no Ψ sites on the CDS region of mTOR (32). The abundance of Ψ was second only to m6A (Ψ/U 0.2-0.4%) in mRNA (33). HCC is a phenotypically and genetically heterogeneous tumor and its tumorigenesis is driven by a variety of molecular mechanisms. Among them, the mTOR pathways, as a central regulator of cell growth and metabolism in response to growth factors and cellular stress, play a key role in regulating HCC development and progression (34). Equally important, post-translational modifications (PTMs) are essential biochemical reactions that covalently regulate the conformation, activity, and stability of proteins, and play a critical role in a broad spectrum of biological processes (35). As an important PTM, protein phosphorylation is involved in the regulation of almost all biological processes in eukaryotes (36). Our study showed that the protein expression of p-mTOR had a positive correlation with PUS1 expression, which indicated that PUS1 promoted tumorigenesis and progression of HCC by regulating mTOR PTMs. It was recently reported that the kinase activity of MTOR modified nearly half of the phosphorylation sites of human ULK1, which was the mostly hyperphosphorylated protein among all ATGs (37). Moreover, the transcription factor and oncoprotein MYC are potent drivers of many human cancers and can regulate numerous biological activities that contribute to tumorigenesis, and they also play an important role in the development of HCC (38). Interestingly, recent study showed that MYC and mTOR are synchronously involved in the regulation of HCC occurrence and progression (39). Our results revealed that PUS1 promotes tumorigenesis and progression of HCC, which is dependent on mTOR and MYC pathways.

## 5 Conclusion

PUS1 could be a novel effective therapeutic target for improving clinical diagnosis, progression surveillance, and prognosis assessment of HCC. PUS1 promotes tumorigenesis, and progression of HCC is dependent on the mTOR and MYC pathways.

## Data Availability

The datasets presented in this study can be found in online repositories. The names of the repository/repositories and accession number(s) can be found in the article/**Supplementary Material**.
