# Supplementary material for: Pseudouridine synthase 1 promotes progression of hepatocellular carcinoma via mTOR and MYC signaling pathways
Source: Front Oncol. 2025 Mar 18;15:1576651. doi: 10.3389/fonc.2025.1576651 (PMC11959026; doi:10.3389/fonc.2025.1576651)

***Supporting Information*** for

**Pseudouridine synthase 1 promotes progression of hepatocellular carcinoma via *mTOR* and *MYC* signaling pathways**

**Table 1:** List of primer.

| Gene Name | Forward Primer | Reverse Primer |
| --- | --- | --- |
| PUS1 | CACGGGCGGGTTTAACTCCAAG | GCTCAGGCGGTAGGTCTCATCC |
| EIF4G2 | GGACAAAGCCCTAGAAGAGCC | GCTGCTGGGCCATCAAAGT |
| GSPT1 | TGCTGGCAAGTCAACCATTGGAG | AGGCCCAAGACAAGTACCAAGTTTC |
| HNRNPC | ATTGTGGGCTGCTCTGTTCATAAGG | ATTGTGGGCTGCTCTGTTCATAAGG |
| DHCR24 | CTGCCGCTCTCGCTTATCTTCG | CTTGCTACCCTGCTCCTTCCATTC |
| WDR43 | CAATAGCAGTGTCAGTTCCCTA | CCCATAGTTTGATTGTTCGACC |
| SCD | AGCTCCTATACCACCACCACCAC | GGCATCGTCTCCAACTTATCTCCTC |
| HNRNPA2B1 | GCTTAAGCTTTGAAACCACAGA | CTTGATCTTTTGCTTGCAGGAT |
| IDH1 | AGCAGTACAAGTCCCAGTTTGAAGC | GCCTCCCTCTGATTTCATAGCTTGG |
| ACSL3 | GTCAGAAACCAAAGACCAACATCGC | CATGAACAATGGCTGGACCTCCTAG |
| HMGCS1 | GCTCTTGGGATGGACGGTATGC | ACTGCTCCAACTCCACCTGTAGG |
| DHFR | GAGAACTCAAGGAACCTCCACAAGG | CAGAACTGCCACCAACTATCCAGAC |
| IARS1 | CATATCCAGTTTCTCCATCGGA | TGGATTTTCCAGGAGCAATACT |
| XPO1 | TATGAGGAATGTCGCAGATACG | GCAAGAATTGATGCATGCATTG |
| ACACA | GCTAACAATGGCATTGCAGCAGTG | TCAGGTGTGACCATGACAACGAATC |
| pre-MYC | TTCCCCTACCCTCTCAACGA | GTGGCCCGTTAAATAAGCTGC |
| MYC | CGGGTAGTGGAAAACCAGCCT | GTGTGACCGCAACGTAGGA |
| pre-mTOR | CTGATGCGGATCTTCCGAGA | GGGGCACAGAGAATGCACAA |
| mTOR | CGAGGGCAGCAACAGTGAGAG | AAGGAGATGGAACGGAAGAAGCC |

**Table 2:** The detail of survival analysis on the 13 PUSs

| RNAseq ID | Expression range of the probe | Cutoff value used in analysis |
| --- | --- | --- |
| 80324 -PUS1, 83480-PUS3, 54517 -PUS7, 150962-PUS10, 83448 PUS7L, 126789-PUSL1, 142940-TRUB1, 26995 -TRUB2, 1736-DKC1, 113000-RPUSD1, 27079 RPUSD2, 285367-RPUSD3, 84881 -RPUSD4 | OS:442 - 2158; DSS: 442 - 2158; PFS: 442 - 2158; RFS: 442 - 1832 | OS:714.92; DSS:: 758.15; PFS: 728.85; RFS: 728.85 |

**Table 3:** The detail of PUS1 survival analysis

| RNAseq ID | Expression range of the probe | Cutoff value used in analysis |
| --- | --- | --- |
| 80324-PUS1 | OS:294 - 5608; DSS: 294 - 5608; PFS: 294 - 5608; RFS: 297 - 5608 | OS:1000; DSS:: 1014; PFS: 1009; RFS: 1461 |


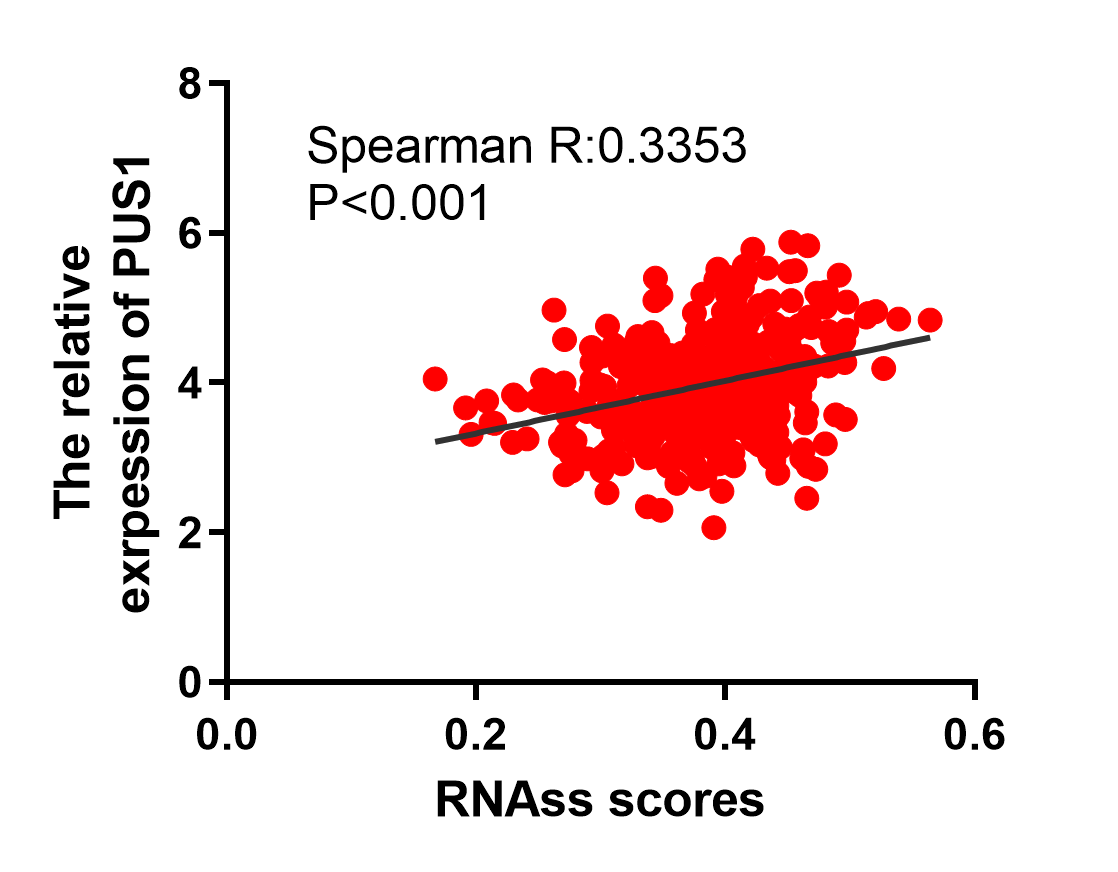


Figure S1. An analysis of the correlation between PUS1 and tumor stemness in HCC.


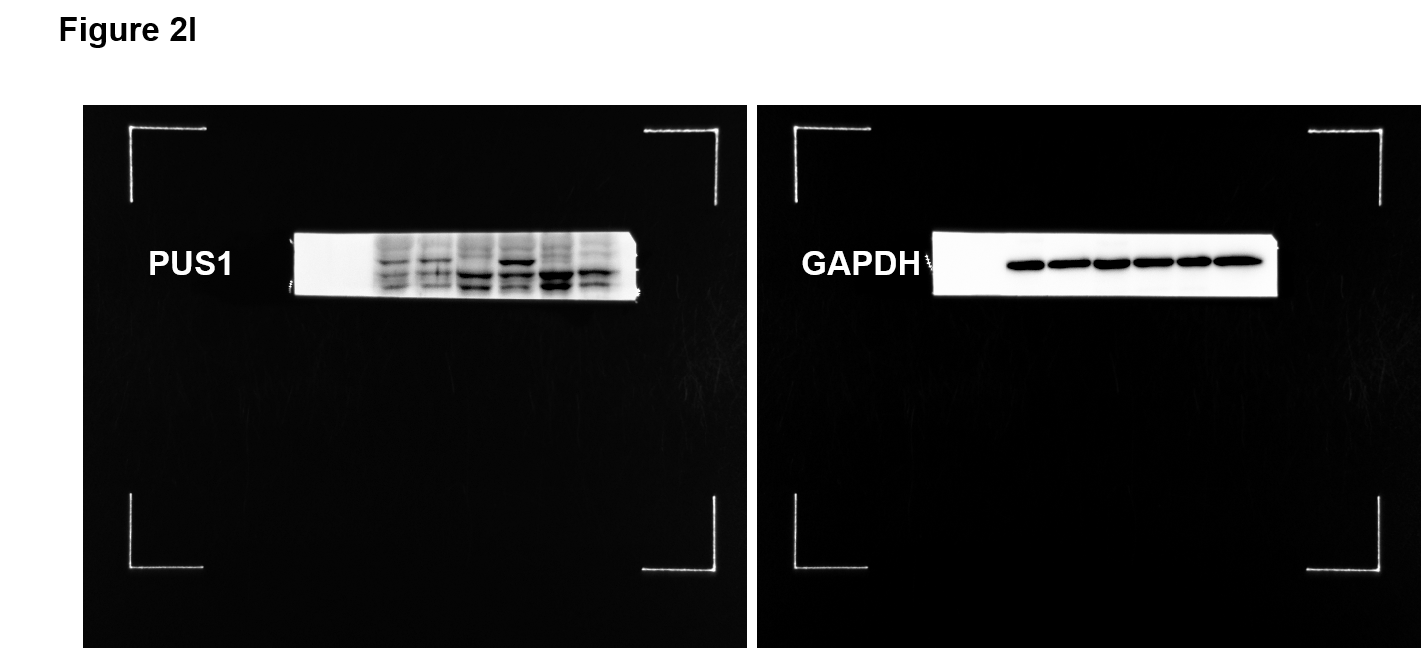


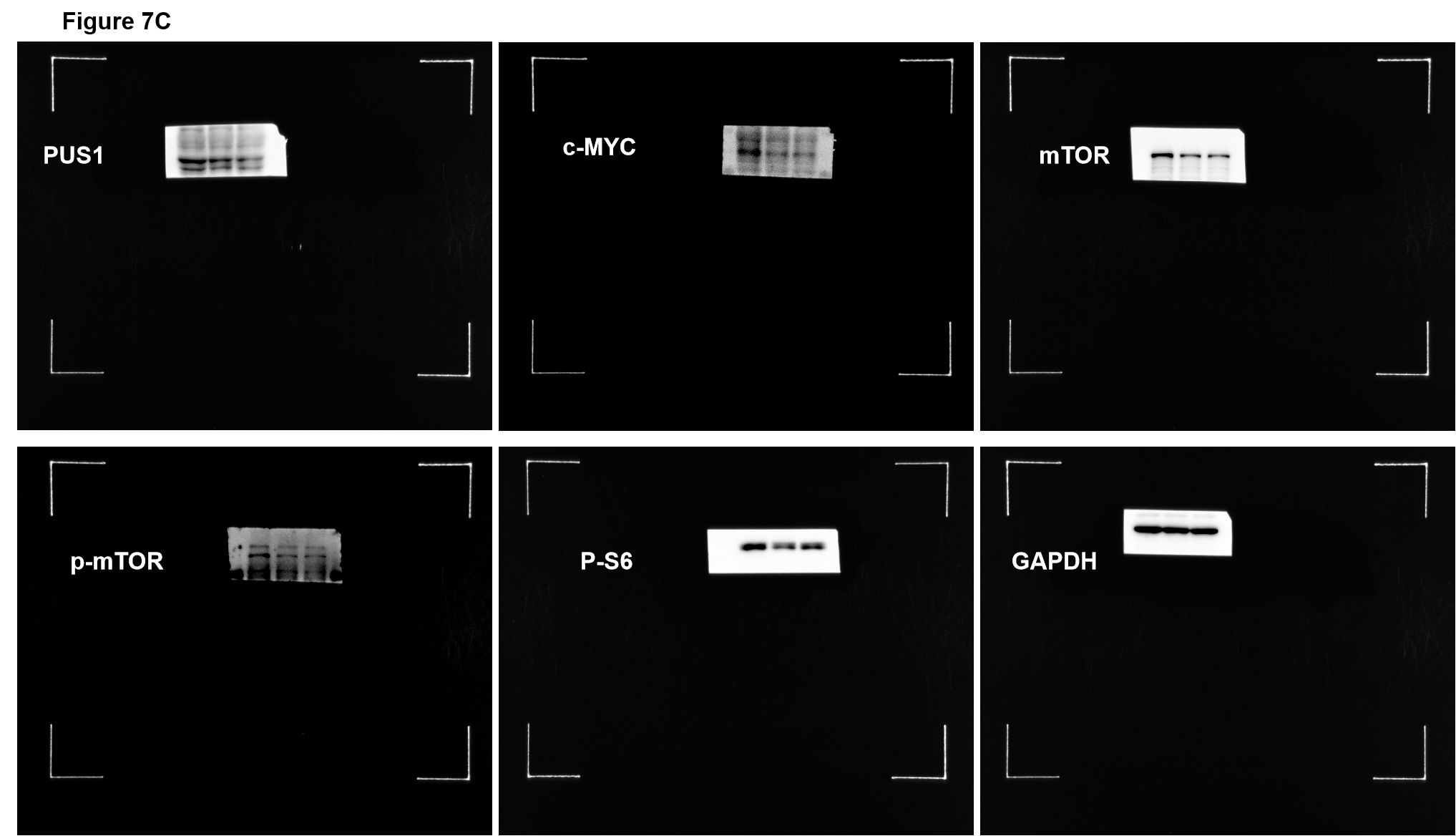


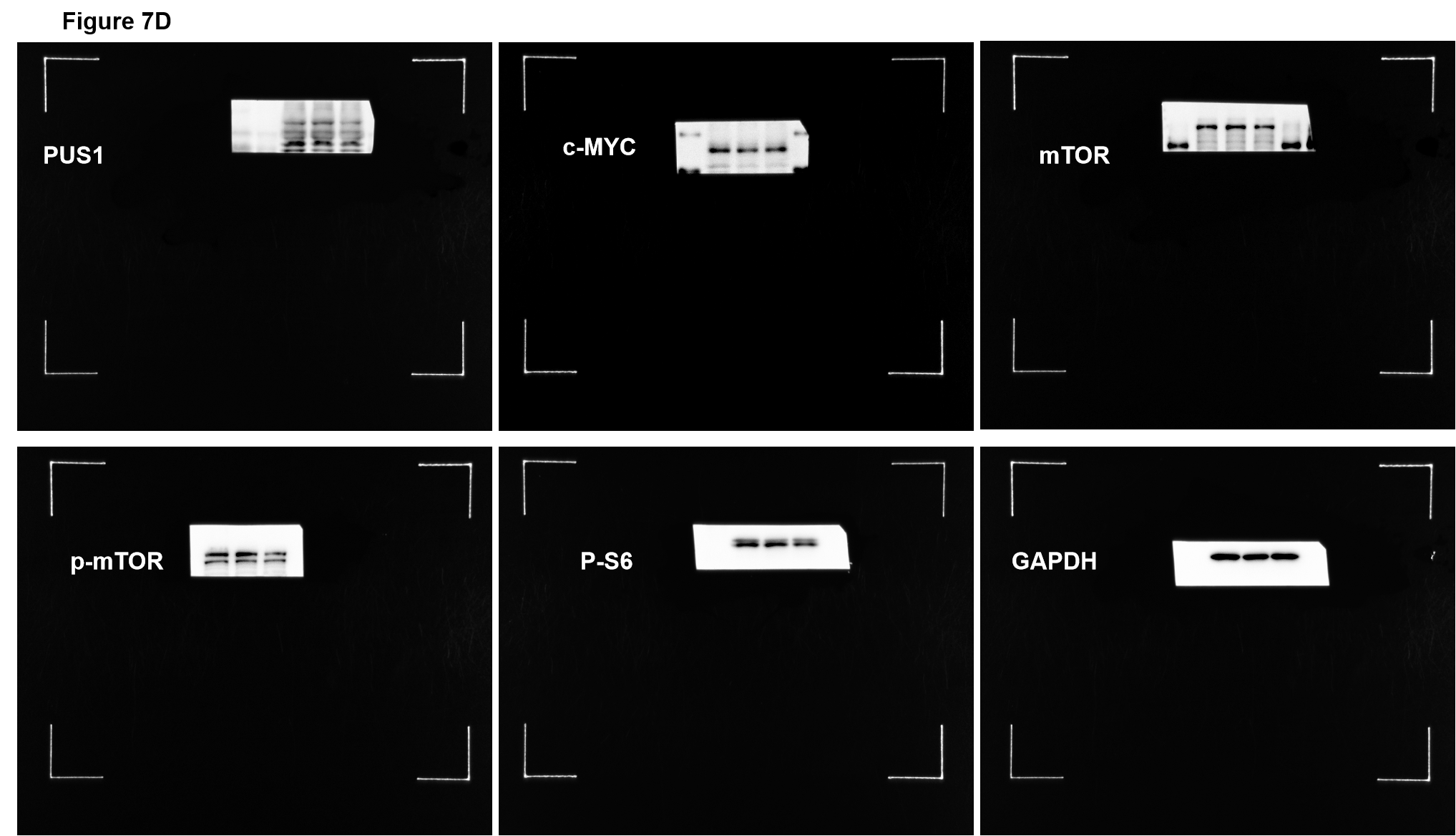

Supplement: Supplementary file 1 [file Table1.docx]
